# [Retracted] STK39, overexpressed in osteosarcoma, regulates osteosarcoma cell invasion and proliferation

**DOI:** 10.3892/ol.2024.14510

**Published:** 2024-06-17

**Authors:** Tao Huang, Yuan Zhou, Yun Cao, Jie Tao, Zhi-Hui Zhou, Dong-Hua Hang

Oncol Lett 14: 4599–4604, 2017; DOI: 10.3892/ol.2017.6728

Following the publication of this paper, it was drawn to the Editor's attention by a concerned reader that certain of the Transwell invasion assay data shown in Fig. 4B on p. 4602 were strikingly similar to data appearing in different form in other articles written by different authors at different research institutes. Owing to the fact that the contentious data in the above article had already been published prior to its submission to *Oncology Letters*, the Editor has decided that this paper should be retracted from the Journal. The authors were asked for an explanation to account for these concerns, but the Editorial Office did not receive a reply. The Editor apologizes to the readership for any inconvenience caused.

